# Long-Term Outcomes of Surgical Aortic Valve Replacement in Patients with Rheumatoid Arthritis

**DOI:** 10.3390/jcm10112492

**Published:** 2021-06-04

**Authors:** Markus Malmberg, Antti Palomäki, Jussi O. T. Sipilä, Päivi Rautava, Jarmo Gunn, Ville Kytö

**Affiliations:** 1 Heart Center, Turku University Hospital and University of Turku, 20520 Turku, Finland; jarmo.gunn@tyks.fi (J.G.); ville.kyto@utu.fi (V.K.); 2 Centre of Rheumatology and Clinical Immunology, Division of Medicine, Turku University Hospital, 20520 Turku, Finland; antti.palomaki@tyks.fi; 3 Department of Medicine, University of Turku, 20520 Turku, Finland; 4 Institute for Molecular Medicine Finland, FIMM, HiLIFE, University of Helsinki, 00290 Helsinki, Finland; 5 Department of Neurology, North Karelia Central Hospital, Siun Sote, 80210 Joensuu, Finland; jussi.sipila@utu.fi; 6 Clinical Neurosciences, University of Turku, 20520 Turku, Finland; 7 Department of Public Health, University of Turku, 20520 Turku, Finland; paivi.rautava@tyks.fi; 8 Turku Clinical Research Centre, Turku University Hospital, 20520 Turku, Finland; 9 Research Center of Applied and Preventive Cardiovascular Medicine, University of Turku, 20520 Turku, Finland; 10 Center for Population Health Research, Turku University Hospital and University of Turku, 20520 Turku, Finland; 11 Administrative Center, Hospital District of Southwest Finland, 20520 Turku, Finland; 12 Department of Public Health, Faculty of Medicine, University of Helsinki, 00014 Helsinki, Finland

**Keywords:** aortic valve stenosis, surgical aortic valve replacement, cohort study, outcomes, rheumatoid arthritis

## Abstract

**Background:** Patients with rheumatoid arthritis (RA) have increased risk of developing cardiovascular disease and events. Little is, however, known about the influence of RA to the outcomes after surgical aortic valve replacement (SAVR). **Methods:** In a retrospective, nationwide, multicenter cohort study, RA patients (n = 109) were compared to patients without RA (n = 1090) treated with isolated SAVR for aortic valve stenosis. Propensity score-matching adjustment for baseline features was used to study the outcome differences in a median follow-up of 5.6 years. **Results:** Patients with RA had higher all-cause mortality (HR 1.76; CI 1.21–2.57; *p* = 0.003), higher incidence of major adverse cardiovascular events (HR 1.63; CI 1.06–2.49; *p* = 0.025), and they needed more often coronary artery revascularization for coronary artery disease (HR 3.96; CI 1.21–12.90; *p* = 0.027) in long-term follow-up after SAVR. As well, cardiovascular mortality rate was higher in patients with RA (35.7% vs. 23.4%, *p* = 0.023). There was no difference in 30-day mortality (2.8% vs. 1.8%, *p* = 0.518) or in the need for aortic valve reoperations (3.7% vs. 4.0%, *p* = 0.532). **Conclusions:** Patients with rheumatoid arthritis had impaired long-term results and increased cardiovascular mortality after SAVR for aortic valve stenosis. Special attention is needed to improve outcomes of aortic valve stenosis patients with RA after SAVR.

## 1. Introduction

Rheumatoid arthritis (RA) is a chronic autoimmune inflammatory disease affecting primarily the joints but with frequent systemic and extra-articular manifestations [1]. In these patients, cardiovascular disease has been recognized as a major cause of increased morbidity and mortality [2]. Valvular abnormalities including granulomatous involvement resembling rheumatoid nodules along with valvular calcification and thickening are more frequently observed in patients with RA [3,4,5,6]. However, there is currently very limited evidence regarding RA patients treated with valvular surgery and especially with aortic valve stenosis. Previous studies have indicated that RA does not increase in-hospital mortality after transapical (TAVR) or surgical aortic valve replacement (SAVR) [7,8], but there are no studies about the long-term results of these procedures on RA patients. Therefore, we investigated the outcomes of SAVR for aortic valve stenosis in patients with RA.

## 2. Patients and Methods

### 2.1. Study Design and Population

**Figure 1:**
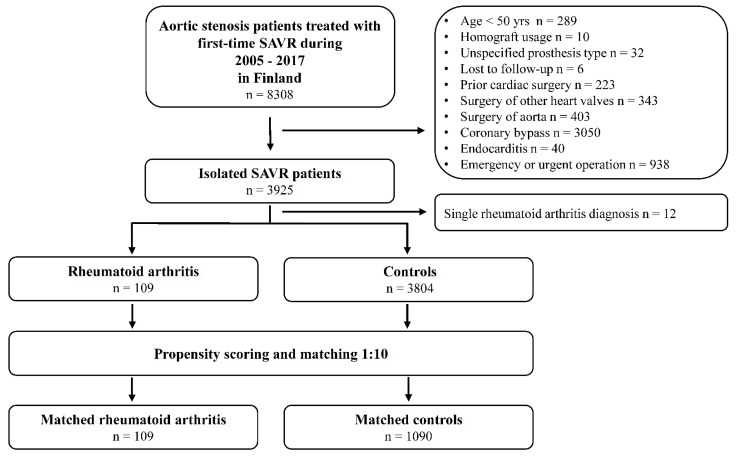
Study flow chart. SAVR = Surgical aortic valve replacement.

Aortic valve stenosis patients treated with first-time isolated SAVR between 1 June 2005 and 31 December 2017 (n = 8308) were retrospectively recognized from the Care Register for Healthcare in Finland. This mandated-by-law, nationwide registry includes data on all hospital admissions and major surgical procedures in Finland [9]. SAVR was performed in eight hospitals (two private and six public) during the study period. Patients aged <50 years, patients with prior cardiac surgery, endocarditis, emergency operation, concomitant surgery of other heart valves, aorta, or coronary artery bypass, patients treated with aortic valve homograft, and patients with unspecified prosthesis type were excluded from the study. Six patients were lost to follow-up. Patients with RA were recognized from the database using ICD-10 codes M05 and M06 for seropositive and seronegative RA, respectively. To increase the specificity of RA diagnosis, only RA patients with at least two episodes with RA diagnosis in specialized care were included, resulting in 109 patients with and 3804 without RA (Figure 1).

### 2.2. Definitions

The primary outcome of interest was 10-year all-cause mortality. Secondary outcomes were major adverse cardiovascular event (MACE; defined and myocardial infarction, stroke, or cardiovascular death), aortic valve related reoperation, and coronary artery revascularization within 10 years from primary surgery. In addition, 5-year interim analyses were performed. The outcomes were recognized from admission records and death certificates (Supplement methods). Comorbidities were recognized from the Care Register for Healthcare and the Finnish Cancer Registry using previously described ICD coding [10]. Mortality data were obtained from the nationwide cause of death registry held by Statistic of Finland. Follow-up ended on 31 December 2018. 

### 2.3. Matching and Statistical Analysis

Propensity score based on baseline characteristics (Table 1) was created using logistic regression. Included variables were considered relevant based on literature review and clinical judgement. Patients with non-overlapping propensity scores were excluded (n = 255 patients without RA). Trimmed propensity score was used for local optimal 1:10 caliber matching without replacing using 0.10 caliper width of the logit of standard deviation [11]. Unmeasured confounding was estimated by calculating the E-value as previously described [12]. Effect sizes of baseline characteristics between study groups were evaluated by standardized mean difference scores (SMD). Outcomes were studied with Kaplan–Meier method and Cox regression. Matched regression models were used in analysis of propensity-matched groups. In addition, multivariable Cox models adjusted with the same variables used for propensity scoring were studied in the non-matched cohort. Influence of prosthetic valve type on associations between RA status and outcomes was studied with interaction-term analysis. Association of seropositivity with long-term mortality was studied using Cox regression in RA patients adjusted for baseline features (Table 1). Proportional hazard assumptions were examined by visual examination of Schoenfeld residuals. Cause-specific hazard models for competing risk due to death were applied in analysis of other long-term outcomes. Results are given as the mean, median, percentage, or hazard ratio (HR) with 95% confidence intervals (CI). *p* value < 0.05 was inferred statistically significant. Analyses were performed with SAS version 9.4 (SAS Institute Inc., Cary, NC, USA).

## 3. Results 

Of all included aortic valve stenosis patients (median age 72 years, 48.7% women), 2.8% had RA. Patients with RA were older, more frequently women, and had higher proportion of pulmonary disease than SAVR patients without RA (Table 1). RA patients received more often biological aortic valve prosthesis. Propensity matching (1:10) identified 109 RA and 1090 control patients with comparable baseline features (Table 1). The majority of RA patients (85.3%) were seropositive. Median follow-up time for survivors was 5.6 years (min 1.05, max 10.0) with no difference between study groups (*p* = 0.703). 

### 3.1. Mortality

**Figure 2:**
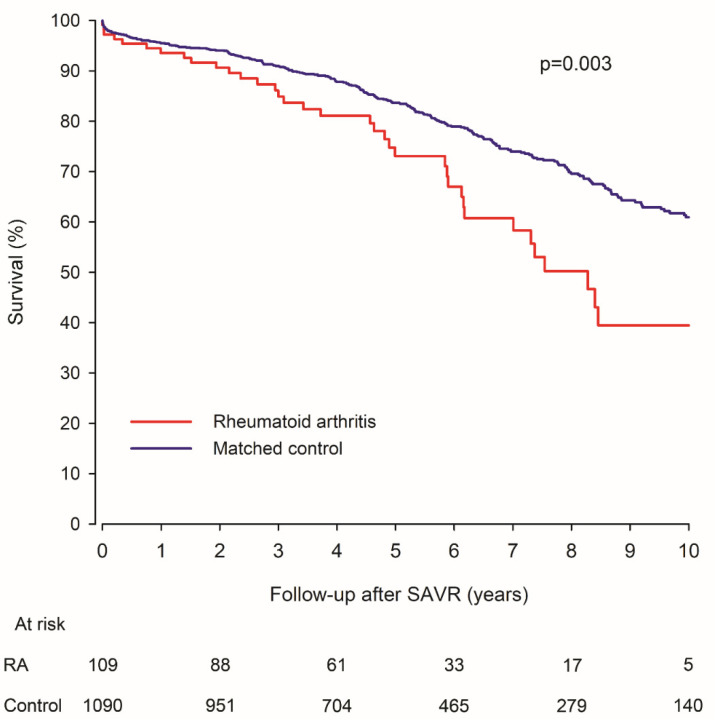
Survival of surgically treated aortic valve stenosis patients with rheumatoid arthritis and matched control patients. RA = Rheumatoid arthritis. SAVR = Surgical aortic valve replacement.

During the follow-up, there were a total of 292 deaths (36 in the RA group). Thirty-day mortality in operated patients was 2.8% in the RA group vs. 1.8% in controls (*p* = 0.518). After the early post-operative period, the survival of RA patients and non-RA patients differentiated increasingly with higher long-term mortality in the RA group (Figure 2). Five-year mortality was 26.9% in the RA group and 16.4% in controls (*p* = 0.038). The mortality rate at the end of the 10-year follow-up was after SAVR was 60.5% in RA patients and 39.1% in controls (HR 1.76; CI 1.21–2.57; *p* = 0.003). The E-value was 2.92 (CI: 1.71–4.58). Type of prosthetic valve did not have a significant influence on association of RA with higher long-term mortality (interaction *p* = 0.150). Seropositivity was not associated with long-term mortality in RA patients in univariate (HR 0.97; *p* = 0.095) or baseline adjusted analysis (HR 1.42; *p* = 0.510). Results of multivariable regression models were comparable to those of the propensity-matched cohort (Supplement Table S1).

### 3.2. MACE

**Figure 3:**
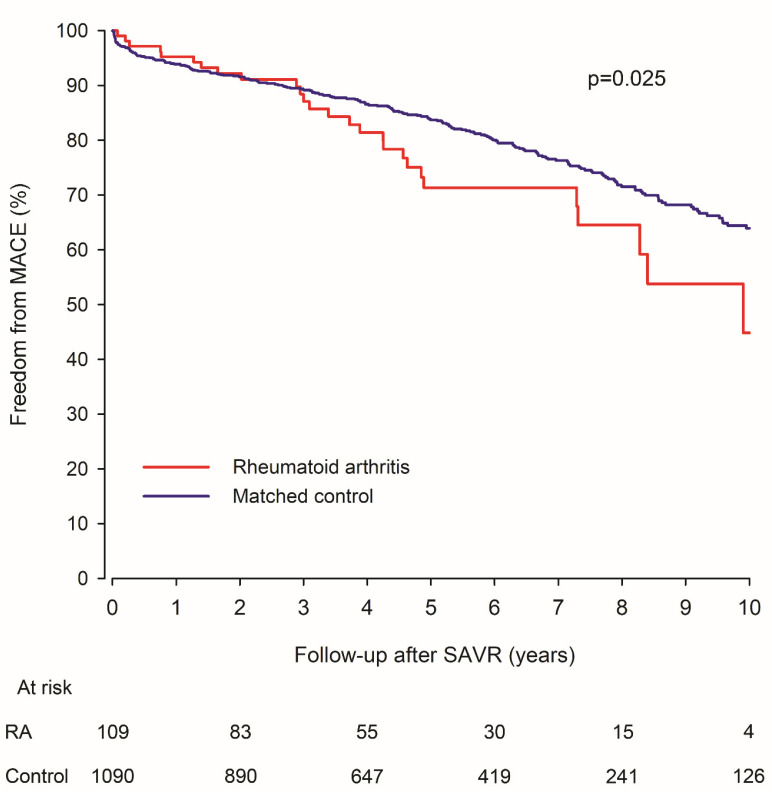
Freedom from major adverse cardiovascular event (MACE) in surgically treated aortic valve stenosis patients with rheumatoid arthritis and matched control patients. RA = Rheumatoid arthritis. SAVR = Surgical aortic valve replacement.

Major adverse cardiovascular event occurred to 255 (27 in the RA group) during the follow-up. RA and control patients had comparable MACE rates up to three years after SAVR, but thereafter the occurrence of MACE continues to increase at a higher rate in RA patients (Figure 3). At 5-year follow-up, 28.6% of RA patients and 16.3% of controls have had MACE (*p* = 0.041). The cumulative MACE rate at 10-year follow-up was 55.2% in RA patients vs. 36.1% in matched controls (HR 1.63; CI 1.06–2.49; *p* = 0.025). The stroke rate during follow-up was 19.1% in RA and 16.8% in control patients (*p* = 0.288). The myocardial infarction (MI) rate during follow-up was 24.1% in RA patients and 8.2% in controls during follow-up (*p* = 0.093). The cardiovascular mortality rate was 35.7% in RA and 23.4% in control patients (HR 1.94; CI 1.10–3.43; *p* = 0.023). Results of the five-year interim analyses are presented in Supplementary Table S2. The type of prosthetic valve did not have significant interaction with association of RA and MACE, stroke, MI, or cardiovascular death (interaction *p* > 0.580 for all). Seropositivity was not associated with MACE or its components in RA patients in (*p* > 0.810, in both univariate and multivariate analysis).

### 3.3. Reoperation

Aortic valve-related reoperation was performed (cumulatively) to 3.7% of RA patients and to 4.0% of control patients during the follow-up (*p* = 0.532). The type of prosthetic valve did not influence the lack of association between RA aortic valve-related reoperation (interaction *p* = 0.992). The majority of the reoperations (76.9%) were surgical aortic valve reoperations. During the follow-up, coronary artery revascularization was performed to 10.3% of RA patients and to 4.3% of control patients who had no need for concomitant coronary artery bypass grafting surgery in the index procedure (HR 3.96; CI 1.21–12.90; *p* = 0.027). The type of prosthetic valve did not influence the associations between RA and aortic valve reoperation or revascularization (interaction *p* = 0.992 and 0.996 respectively). Seropositivity was not associated with reoperations or revascularization in RA patients (*p* > 0.797 in both univariate and multivariate analysis). 

## 4. Discussion

This nationwide multicenter retrospective cohort study shows that patients with RA had increased mortality, greater incidence of MACE, and needed more often coronary artery revascularization during 10-year follow-up after isolated SAVR for aortic valve stenosis. In addition, cardiovascular death was more common in patients with RA.

The current evidence regarding outcomes after aortic valve replacement in patients with RA is limited. Rudasill et al. studied the short-term outcomes after TAVR in patients with connective tissue diseases, resulting with a lower 30-day mortality, but the risks for post-operative infection and septicemia were increased [7]. In their cohort of 2557 patients, 75.6% had RA [7]. In a report by Elbadawi et al., in-hospital mortality was studied after TAVR and SAVR in patients with RA, finding no difference between the two treatment options [8]. To our knowledge, our report was the first study investigating the long-term outcomes of RA patients treated with SAVR for aortic valve stenosis. 

In studies regarding mitral valve, Stulak et al. compared 36 patients with RA to 72 patients without RA after mitral valve repair in a median follow-up of 4.2 years [13]. In their study, patients with RA had decreased survival (27% vs. 64%, *p* = 0.005) and increased risk for reoperation (freedom from reoperation 93% vs. 98%, *p* = 0.04) [13]. In a study by Vassileva et al., the researchers did not find a difference in in-hospital mortality after mitral valve surgery in patients with RA [14]. These results highlight the need for long-term follow-up studies in order to have more accurate data on this entire patient cohort. 

Overall, valvular heart diseases are more common in patients with RA, but they are often mild and asymptomatic [3,5,15]. In a follow-up study of patients with mild and moderate aortic valve stenosis, the progression rate to more severe stenosis was not increased in patients with RA when compared to general population [16]. As the incidence of aortic stenosis in highly age-dependent [17], also RA patients presenting with symptomatic aortic valve stenosis are typically elderly with multiple comorbidities, as our study indicated.

In our study, we found out that patients with RA had higher cardiovascular mortality and needed more often revascularization for coronary artery disease in long-term. Therefore, it is possible that our results might be explained at least in part by the higher incidence of coronary artery disease in the RA cohort developed during the post-operative follow-up period. Moreover, there seems to be no difference in valve-related reoperations indicating a similar rate of structural valve degeneration, although the majority (80%) of all implanted valves where bioprostheses. It has not been clearly indicated that RA would act as an independent risk factor for early structural valve degeneration, although inflammation itself seems to play a key role in destruction of heart valves [18,19].

Higher revascularization rate in our study indicates that the development of symptomatic cardiovascular disease is more common after SAVR in these patients. As we know from previous studies, the most common cardiac manifestations of RA are associated with atherosclerosis [20], with an increased risk of MI and cardiovascular death [2,21], making the prevention of atherosclerosis one of the key priorities in the treatment of RA patients [22]. Fortunately, there are signs that the excess cardiovascular mortality in patients with RA has decreased significantly during recent decades [23,24], possibly because of more safe and efficient RA therapies and more focus on cardiovascular prevention [25]. The prognosis of symptomatic aortic valve stenosis is extremely poor without treatment [26,27]. Thus, importantly, our results should not be interpreted in the way that would result in exclusion of RA patients from interventional treatment for aortic valve stenosis.

Our study has limitations. First, the retrospective nature of the study limits the possibility to interpret the results. The data that we used were collected from reliable registries, mandated by law in Finland, but it is, however, possible that sources of bias are present [28]. Coding errors are possible since diagnoses were determined by clinicians. The incidence of RA patients in this study (2.8%) is comparable to previously reported in SAVR population (4%) [6]. Second, more detailed information regarding in-hospital or operative data, RA disease activity or duration, pharmacotherapy of RA or cardiovascular diseases, or laboratory results were not available due to nature of available registries. Pharmacotherapies have also evolved during the 12-year recruitment period. However, it is not expected that this would have significant impact to the major findings of this study. Third, to balance the differences between the study groups, we used propensity-score matching. Although it is one of the strongest control cofounding factors, it is still possible that unrecognized residual co-founders may impact the results. Based on the E-value, the observed higher all-cause mortality in RA patients could be explained away by an unmeasured confounder that was associated with both RA and the outcome by a risk ratio of 2.9-fold each, above and beyond the measured confounders, but weaker confounding could not do so [12]. Fourthly, discovering the mediators of poorer outcome in RA patients were beyond the current study.

In conclusion, patients with RA are at higher risk of long-term all-cause mortality and MACE and need more often coronary artery revascularization after SAVR for aortic valve stenosis than patients without RA. Special attention and further studies are required to improve the outcomes of this vulnerable patient population. 

## Figures and Tables

**Table 1:** Baseline features of rheumatoid arthritis and control patients with isolated surgical aortic valve replacement for aortic stenosis. All and propensity score matched patients. SMD = Standardized mean difference.

	Original Cohort			Matched Cohort		
	Rheumatoid Arthritis	Control		Rheumatoid Arthritis	Control	
Variable	n = 109	n = 3804	|SMD|	n = 109	n = 1090	|SMD|
Age, years (SD)	71.7 (8.0)	70.7 (8.7)	0.13	71.7 (8.0)	71.8 (8.4)	0.02
Female sex	70 (64.2%)	1835 (48.2%)	0.33	70 (64.2%)	685 (62.8%)	0.07
Comorbidities						
Atrial fibrillation	22 (20.2%)	863 (22.7%)	0.06	22 (20.2%)	235 (21.6%)	0.08
Cerebrovascular disease	13 (11.9%)	391 (10.3%)	0.05	13 (11.9%)	128 (11.7%)	0.01
Chronic pulmonary disease	22 (20.2%)	439 (11.5%)	0.24	22 (20.2%)	209 (19.2%)	0.03
Diabetes	12 (11.0%)	640 (16.8%)	0.17	12 (11.0%)	120 (11.0%)	0.01
Heart failure	27 (24.8%)	890 (23.4%)	0.03	27 (24.8%)	235 (21.6%)	0.08
Hypertension	45 (41.3%)	1683 (44.2%)	0.06	45 (41.3%)	507 (46.5%)	0.09
Malignancy	17 (15.6%)	451 (11.9%)	0.11	17 (15.6%)	158 (14.5%)	0.01
Peripheral vascular disease	6 (5.5%)	210 (5.5%)	0.001	6 (5.5%)	64 (5.9%)	0.004
Psychotic disorder	1 (0.9%)	32 (0.8%)	0.01	1 (0.9%)	6 (0.6%)	0.02
Prior myocardial infarction	8 (7.3%)	259 (6.8%)	0.02	8 (7.3%)	69 (6.3%)	0.04
Renal failure	1 (0.9%)	73 (1.9%)	0.09	1 (0.9%)	10 (0.9%)	0.05
Type of aortic valve prosthesis			0.33			0.06
Biological	87 (79.8%)	2727 (71.7%)		87 (79.8%)	902 (82.8%)	
Mechanical	22 (20.2%)	1077 (28.3%)		22 (20.2%)	188 (17.3%)	
Surgical center (n = 8)			0.10			0.03

## Data Availability

The data and study materials will be made available to those who fulfill requirements of applicable Finnish laws and regulations for purposes of reproducing the results or replicating the procedure (from the corresponding author).

## Supplementary Materials

The following are available online at https://www.mdpi.com/article/10.3390/jcm10112492/s1, Table S1: Results of unadjusted and multivariable adjusted Cox-models, Table S2: Five-year interim outcome results.

## Abbreviations

RA	Rheumatoid arthritis
CAD	Coronary artery disease
TAVR	Transcatheter aortic valve replacement
SAVR	Surgical aortic valve replacement
MACE	Major adverse cardiovascular event
SMD	Standardized mean difference
HR	Hazard ratio
CI	95% confidence intervals
MI	Myocardial infarction
