# Supplementary material for: Long-Term Outcomes of Surgical Aortic Valve Replacement in Patients with Rheumatoid Arthritis
Source: J Clin Med. 2021 Jun 4;10(11):2492. doi: 10.3390/jcm10112492 (PMC8200235; doi:10.3390/jcm10112492)
Supplement: Supplementary file 1 [file jcm-10-02492-s001.zip › jcm-1216545-supplementary.pdf]

Supplement Methods

Data Sources

Following nationwide, mandated-by law registry data was collected from all study patients:

- Data of all hospital and emergency room admissions (International Classification of Diagnosis / ICD-10) and operational codes (Nordic Classification of Surgical Procedures) collected from the CRHF registry held by the National Institute for Health and Welfare of Finland.
- Mortality data including date and causes of death held by the Statistics Finland.

Outcomes

The outcomes of interest were 10-year all-cause mortality, combined major adverse cardiovascular event (MACE; defined as myocardial infarction, stroke, or cardiovascular mortality), aortic valve reoperation, and coronary artery revascularization. MACE, reoperation and revascularization was studied after discharge from index procedure. Follow-up of all endpoints ended Dec 31st 2018.

Definitions

- Aortic stenosis: ICD-10 codes I35.0, I35.2
- Major adverse cardiovascular event: Cardiovascular death, stroke, or myocardial infarction.
- Cardiovascular death: Underlying cause of death ICD-10 code I
- Stroke: ICD-10 code I60-I64.
- Myocardial infarction: ICD-10 codes I21, I22.
- All-cause mortality: Death due to any cause.
- Aortic valve re-operation: Surgical or percutaneous aortic valve procedure, operational codes FMA, FMB, FMC, FMD, FMW, FME, FCA60, FCA70
- Revascularization: Percutaneous coronary intervention or coronary artery surgery. Operational codes FN2AA, FN2AB, FN2BA, FN2CA, FN2CB, FN2CC, FN2CD, FN2CE, FN2CT, FN2DA, FN2DB, FN2EA, FN1AT, FN1BT, FN1YT, FNG00, FNG10, FN2DA, TFN40, TFN50, TFN10, FN2EF, FN2EB, FN2EC, FN1ST, FNA, FNB, FNC, FND, FNE

Supplement Table. year follow-up. MACE = major adverse cardiovascular event.

	Univariable		Multivariable	
**Outcome**	**HR (95%CI)**	***p*-Value**	**HR (95%CI)**	***p*-Value**
**Death**	1.90 (1.36–2.65)	0.0002	1.92 (1.37–2.69)	0.0002
**MACE**	1.46 (1.00–2.15)	0.050	1.48 (1.01–2.19)	0.046
**Myocardial infarction**	1.74 (0.82–3.71)	0.150	1.85 (0.86–3.97)	0.114
**Stroke**	1.17 (0.66–2.07)	0.600	1.20 (0.67–2.14)	0.537
**Cardiovascular death**	1.96 (1.21–3.18)	0.007	1.94 (1.19–3.18)	0.008
**Aortic valve reoperation**	0.54 (0.08–3.90)	0.543	0.59 (0.08–4.30)	0.605
**Revascularization**	5.00 (1.99–12.58)	0.001	5.35 (2.07–13.82)	0.001
